# 877. Care at Burn Centers is Associated with Reduced Discharge Against Medical Advice: A Multi-database Analysis

**DOI:** 10.1093/jbcr/irag033.363

**Published:** 2026-04-06

**Authors:** Jennifer K Shah, Mollie B Smith, Clifford C Sheckter

**Affiliations:** Geisel School of Medicine at Dartmouth, New York, NY; University of Massachusetts T. H. Chan School of Medicine, Worchester, MA; Stanford University School of Medicine, Palo Alto, CA

## Abstract

**Introduction:**

Discharge against medical advice (AMA) leads to worse outcomes in burn patients including higher readmission rates, wound complications, and elevated costs. There is limited understanding of how burn center status and behavioral interventions mitigate AMA discharge. This study aims to answer these questions.

**Methods:**

Burn encounters were identified in two national databases– the Nationwide Inpatient Sample (NIS), 2016–2021, and the Burn Care Quality Platform (BCQP), 2016–2022. Encounters were stratified by AMA discharge. The primary outcome was the effect of inpatient psychosocial behavioral interventions, including substance use counseling, mental health counseling, and social support, which were evaluated with propensity score matching. Additional outcomes included the effects of burn center status on AMA discharge along with the overall incidence of AMA discharge. Burn center status was inferred in the NIS by identifying facilities that admitted at least 100 weighted burn encounters annually. Regression models were adjusted for burn injury characteristics, demographics, primary payer, housing status, mental health comorbidities, and facility characteristics.

**Results:**

In the NIS, 5950 (2.8%) of 214 390 burn encounters were discharged AMA; in the BCQP, 2923 (1.4%) of 205 993 burn encounters were discharged AMA. Median length of stay (LOS) in days (interquartile range) among encounters who did and did not discharge AMA was 2 (1, 5) versus 4 (2, 9), respectively, in the NIS (*p*<.01), and 3 (1, 8) versus 4 (1, 10), respectively, in the BCQP (*p*<.01). There was no effect of psychosocial behavioral intervention on the likelihood of AMA discharge (*p*=.85). Significant predictors of AMA discharge included male sex, Medicaid or self-pay, smaller burns, and more recent year (*p*<.01). The incidence of AMA discharges increased over the study period in both NIS (IRR 1.2; 95% CI: 1.1–1.2; *p*<.01) and the BCQP (IRR 1.2; 95% CI: 1.2–1.2; *p*<.01). Within a propensity-score matched model of encounters in the NIS matched on established risk factors for AMA (age, sex, year, primary payer, substance use disorders, homelessness, and total body surface area (TBSA) of burns), care at a burn center was associated with a 1.2% decrease in AMA discharge (ATE -0.01; 95% CI: -0.02– -0.00; *p*=.01).

**Conclusions:**

The incidence of AMA discharges is increasing. Adjusting for burn severity and established risk factors, receiving care at a burn center significantly decreased AMA discharge. Existing interventions to alleviate AMA discharge may be helpful, but they are insufficient to significantly alter patterns of AMA discharge.

**Applicability of Research to Practice:**

Burn patients at risk for AMA discharge should be treated at burn centers. Additional measures beyond behavioral psychosocial interventions are needed to decrease the growing problem of AMA discharge in burn patients.

**Funding for the study:**

N/A.

